# The Successful Treatment of Acute Kidney Injury Due to Antineutrophil Cytoplasmic Antibody (ANCA)-Associated Vasculitis Without Glomeruli Involvement by Using Rituximab: A Case Report

**DOI:** 10.7759/cureus.50732

**Published:** 2023-12-18

**Authors:** Neeladri Misra, Khalid Mahmood

**Affiliations:** 1 Internal Medicine, Sutter Roseville Medical Center, Roseville, USA

**Keywords:** antineutrophil cytoplasmic antibody (anca) associated vasculitis (aav), vasculitis, prednisone treatment, rituximab therapy, anca associated vasculitis

## Abstract

Antineutrophil cytoplasmic antibody (ANCA)-associated vasculitis (AAV) refers to a group of disorders characterized by inflammation and destruction of small- and medium-sized blood vessels. It can be classified into various clinical disease phenotypes: granulomatosis with polyangiitis (GPA), microscopic polyangiitis (MPA), eosinophilic granulomatosis with polyangiitis (EGPA), and renal-limited AAV or serologic subtypes, which are myeloperoxidase (MPO)-AAV and proteinase 3 (PR3)-AAV. Renal involvement is a common manifestation in these types of vasculitis.

MPO-AAV usually involves the glomeruli causing membranous changes and presents with glomerulonephritis. However, MPO-AAV renal type without glomeruli involvement is much rarer, and very few case reports of this condition have been reported in the literature. Once the diagnosis is confirmed by renal biopsy, the treatment of AAV involves high-dose steroids and cyclophosphamide to induce remission. Rituximab, a chimeric monoclonal antibody that targets against the pan-B-cell marker CD20, was the first monoclonal antibody to be approved for the treatment of vasculitis. It is now considered first-line therapy for ANCA vasculitis with kidney involvement thanks to the higher remission rates associated with it.

We report a unique and rare case of acute kidney injury due to MPO-AAV without glomeruli involvement, which was successfully treated with rituximab over a period of 12 months and led to the remission of the disease in the kidneys.

## Introduction

Antineutrophil cytoplasmic antibody (ANCA)-associated vasculitis (AAV) comprises disorders characterized by inflammation and destruction of small- to medium-sized blood vessels. AAV diseases include granulomatosis with polyangiitis (GPA), microscopic polyangiitis (MPA), eosinophilic granulomatosis with polyangiitis (EGPA), and renal-limited AAV [1,2]. AAV can also be classified into proteinase 3 (PR3)-AAV and myeloperoxidase (MPO)-AAV based on the presence of the specific correlating antibody. AAV is usually associated with kidneys, with >75% of patients manifesting renal involvement characterized by rapidly progressive glomerulonephritis. The global incidence of AAV is 200-400 cases per million people. The pathogenesis of AAV is multifactorial and influenced by genetics, environmental factors, and responses of the innate and adaptive immune system [2,3]. Randomized controlled trials in the past two decades have led to great advancements in the therapy for AAV and transformed AAV from a fatal disease to a chronic, manageable illness with a relapsing course and associated morbidity. 

Renal-limited MPO-AAV involves the glomeruli causing membranous glomerulopathy, which can result in acute kidney injury due to glomerular destruction and necrotizing vasculitis. However, MPO-AAV causing acute kidney injury without glomeruli involvement (glomeruli-sparing) and tubulointerstitial nephritis are much rarer with only a few case reports published in the literature so far [3,4]. We present a rare case of acute kidney injury caused by MPO-AAV without glomeruli involvement, which was successfully treated with rituximab. This report highlights the evolving landscape of AAV therapy. We succinctly outline the clinical scenario, emphasizing the uniqueness of the case and its successful management with rituximab.

## Case presentation

The patient was an 84-year-old female with a past medical history of polymyalgia rheumatica, hyperlipidemia, and allergic rhinitis who presented to our hospital after a routine outpatient lab testing showed evidence of acute kidney injury with a serum creatinine level of 2.51 mg/dL. She had a baseline creatinine of 0.86-0.90 mg/dl on routine labs three months ago. The patient was normally very active and endorsed malaise consisting of fatigue, bilateral leg pain, and numbness in her palms for several days. Additionally, she reported decreased thirst, appetite, and energy level after playing golf several days ago in hot weather. She had initially seen her primary care provider and had been started on prednisone for a presumed exacerbation of polymyalgia rheumatica and her Lipitor had been discontinued. The patient was then referred to get routine labs and found to have blood urea nitrogen (BUN) of 99 mg/dl, creatinine of 2.51 mg/dl, and erythrocyte sedimentation rate (ESR) of 82 mm/hr. Subsequently, she was referred to our ED for evaluation.

The patient's vital signs on admission were as follows - BP: 196/79 mmHg, temperature: 97.6 °F, pulse: 75, RR: 16/min, and SPO_2_: 98% on room air. Her physical exam was unremarkable. Her labs showed BUN of 99 mg/dl and creatinine of 3.04 mg/dL. Other labs included Na of 129 mmol/L, K of 5.6 mmol/L, chloride of 97 mmol/L, CO_2_ of 23 mmol/L, and glucose of 174 mg/dL. Her albumin was low at 2.3 g/dL (it had been 3.4 a few months ago), alkaline phosphatase was elevated at 219, and her CK level was 48. Her urine was positive for trace protein and blood with a pH of 6.

The patient was admitted and started on IV fluid hydration with normal saline with daily lab monitoring. For further workup of her acute kidney injury, she underwent a US retroperitoneum, which showed normal renal morphology and no obstruction. Despite being on IV hydration for 48 hours, there was no improvement in her serum creatinine. We then proceeded to consult Nephrology for further evaluation of the patient. Given her history of polymyalgia rheumatica, additional lab testing was ordered, which showed an elevated C3 complement level of 168 mg/dl (normal range: 84-160 mg/dl ), positive RA level (56 IU/ml, normal range: 0-15 IU/ml) and high titers of MPO-ANCA (338.3 AI, normal level: <1.0 AI) in serum. She had normal levels of CCP antibodies, cryoglobulin, antiphospholipid antibodies, and uric acid. Unfortunately, the patient's creatinine did not improve and ranged from 3.0 to 3.5 mg/dl. Hence, Nephrology recommended a kidney biopsy for a definitive diagnosis.

**Figure 1:**
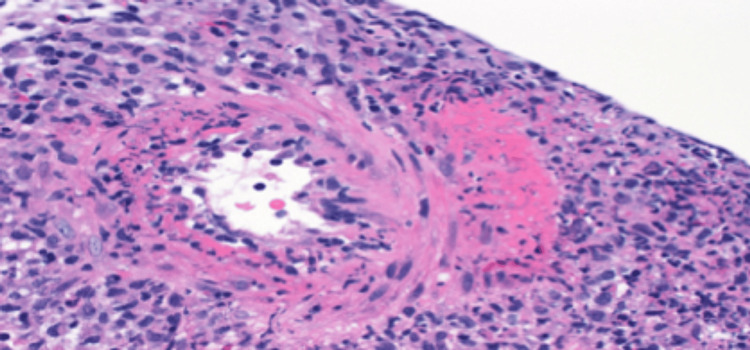
Necrotizing vasculitis in large-caliber vessel

**Figure 2:**
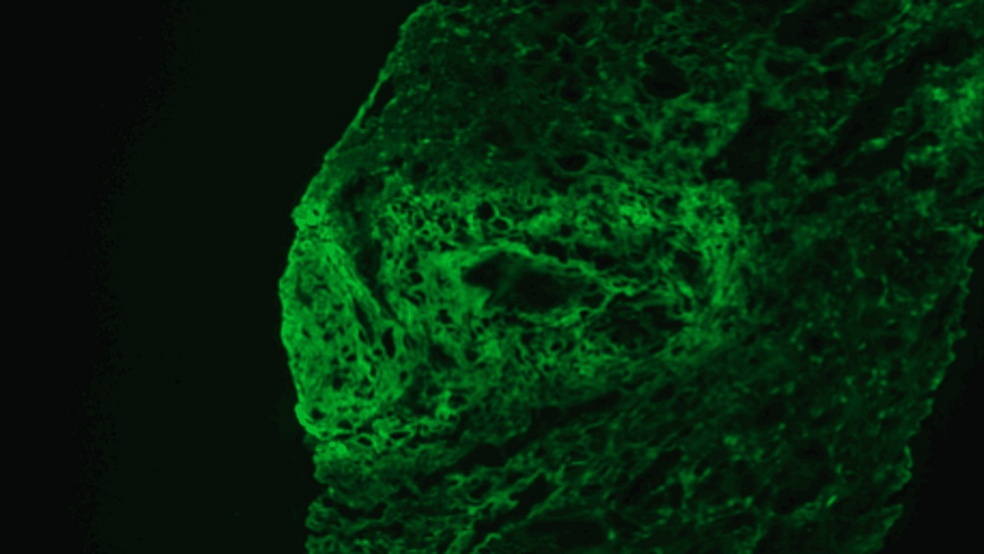
Fibrinogen immunofluorescence

The renal biopsy findings revealed ANCA/MPO-positive pauci-immune necrotizing vasculitis without major glomerular involvement. The biopsy also showed two large-caliber arteries with necrotizing vasculitis, interstitial edema, fibrosis, and atrophy (Figure 1). Immunofluorescence stains on biopsy were negative for immunoglobulin and C3 immunofluorescence staining (Figure 2).

After the biopsy confirmed MPO-AAV without glomeruli involvement, Rheumatology was consulted. Due to the absence of glomeruli involvement, the patient did not have classic MPO-ANCA membranous nephropathy and tubulointerstitial changes. A decision was made to start the patient on rituximab given her MPO-positive status and acute kidney injury. The patient was started on high-dose steroids consisting of prednisone 60 mg and received one dose of 1000 mg rituximab infusion. She tolerated the infusion well and was discharged on a prednisone taper with close follow-up with Nephrology and Rheumatology. Her creatinine level at the time of discharge was 2.88 mg/dl.

The patient showed gradual improvement, with labs showing BUN at 48 mg/dL and creatinine at 2.25 mg/dL one-month post-discharge. Her titers of MPO-ANCA showed improvement and were now at 2.1 AI (normal level: <1 AI, patient value at diagnosis: 338.3). A regimen of rituximab 500 mg every six months and a prednisone taper helped to maintain remission. At the three-month follow-up, renal function was found to have improved further (BUN: 45 mmol/L, creatinine: 1.97 mg/dL), which prompted us to initiate a second rituximab dose at six months. At 12 months, renal function tests revealed a BUN level of 33 mmol/L and creatinine of 1.56 mg/dL, which led to the complete tapering of steroids. The patient remained complication-free, sustaining an active lifestyle on continued rituximab infusions for an additional 12 months.

## Discussion

There are only a few case reports of AAV causing acute kidney injury without glomerular involvement [4-5] and with tubule interstitial changes. Our case highlights the vague clinical manifestations that AAV patients often present with, as well as the diagnostic challenges in the workup for acute kidney injury due to AAV. Acute kidney injury due to AAV is seen in all subtypes of AAV: MPA, GPA, EGPA, and renal-limited AAV [6]. The main organs involved in AAV patients are the kidneys, respiratory tract, and occasionally the heart and the brain. Kidney involvement is common in AAV, especially in GPA and MPA [6], with patients presenting with glomerulonephritis. The diagnosis of acute kidney injury involves renal biopsy, which is considered the gold standard for the diagnosis of AAV affecting kidneys [7].

Outcomes in patients with AAV depend on the time to diagnosis of AAV kidney disease [8], and studies show that older age, lower GFR, and MPO-ANCA positivity are associated with poorer renal outcomes, and the survival rate in MPO ANCA-positive renal disease at 10 years is approximately 45% [9]. High-dose steroids, cyclophosphamide, and azathioprine are the mainstays of therapy for induction as well as remission of AAV involving kidneys [10]. However, even after undergoing this therapy, the rate of progression to end-stage renal disease (ESRD) requiring renal replacement therapy remains high in this patient population [10]. 

Rituximab was first introduced to vasculitis therapy for patients whose condition was refractory to or intolerant of standard agents and had persisting ANCA positivity and was based on the rationale that ANCA contributed to pathogenesis and a B cell-targeted therapy would reduce ANCA levels [11]. Initial studies showed rituximab to be non-inferior to cyclophosphamide [12]. However, since then, several studies have shown it to be better for the treatment of AAV with a superior steroid-sparing effect and higher rates of remission [13]. The 2021 American College of Rheumatology/Vasculitis Foundation guidelines recommend rituximab as first-line therapy for AAV for induction and remission along with the use of corticosteroids [11,14]. Rituximab has emerged as a pivotal therapeutic agent for ANCA vasculitis with renal involvement, displaying significant efficacy, steroid-sparing effects, and higher remission rates. Recent guidelines have also endorsed rituximab as first-line therapy, reinforcing its role in treating vasculitis. Our case was unique as there are very few case reports of MPO-AAV without glomeruli involvement and successful treatment of the condition with rituximab, transforming a potentially fatal condition into a chronic, manageable one [14]. This has made AAV-associated Kidney disease a chronic condition rather than a serious one progressing to ESRD.

## Conclusions

MPO-AAV is a subtype of vasculitis associated with poor prognosis once the kidneys are affected. It often presents as new-onset acute kidney injury with vague symptoms for several weeks to months before a diagnosis can be made, which requires the presence of circulating MPO antibodies and renal biopsy showing necrotizing vasculitis and glomerular changes. Once diagnosed, the treatment should be initiated promptly to prevent the progression of renal disease. Our case involved a unique presentation since the patient's pathology showed vasculitis without glomerular involvement, and hence it did not fit with classic membranous nephropathy as seen in MPO-ANCA renal disease. Some interstitial changes were evident on the biopsy. Treatment was initiated as per the guidelines for MPO-AAV, and rituximab is effective for AAV with renal involvement and associated with high disease-remission rates.

We believe this report contributes to the scientific literature that holds that initiating rituximab in patients presenting with acute kidney injury associated with AAV helps in earlier renal recovery and halts progression to ESRD requiring renal replacement therapy; in our patient, the serum creatinine levels improved after 12 months of therapy with minimal loss of renal function. Furthermore, rituximab is associated with better compliance among patients since it involves six-monthly dosing rather than the daily dosing required with immunosuppressive drugs such as cyclophosphamide. It enables patients to remain in remission and return to normal activities of daily life as seen in our case where the patient had led an active lifestyle before the disease and, post rituximab therapy, has continued to remain active with minimal disruption.
